# The Predictive Role of [18F]FDG PET/CT in Early HCC Recurrence After Liver Transplantation

**DOI:** 10.3390/cancers18040555

**Published:** 2026-02-09

**Authors:** Eleonora Alimenti, Lorenzo Canova, Massimo Iavarone, Giovanni Aldinio, Daniele Dondossola, Luigia Florimonte, Eloisa Franchi, Giulia Marini, Clara Dibenedetto, Lucio Caccamo, Federica Cerini, Massimo Castellani, Cristiano Quintini, Pietro Lampertico

**Affiliations:** 1 Division of Gastroenterology and Hepatology, Foundation IRCCS Ca’ Granda Ospedale Maggiore Policlinico, Via F Sforza 28, 20122 Milan, Italy; eleonora.alimenti@policlinico.mi.it (E.A.); giovanni.aldinio@policlinico.mi.it (G.A.); clara.dibenedetto@policlinico.mi.it (C.D.); pietro.lampertico@unimi.it (P.L.); 2 Division of Hepatology, San Giuseppe Hospital, IRCCS MultiMedica, 20122 Milan, Italy; lorenzo.canova@multimedica.it (L.C.); federica.cerini@unimi.it (F.C.); 3 CRC “A. M. and A. Migliavacca” Center for Liver Disease, Department of Pathophysiology and Transplantation, University of Milan, 20122 Milan, Italy; 4 General and Liver Transplant Surgery Unit, Foundation IRCCS Ca’ Granda Ospedale Maggiore Policlinico, 20122 Milan, Italy; daniele.dondossola@policlinico.mi.it (D.D.); eloisa.franchi@policlinico.mi.it (E.F.); giulia.marini@unimi.it (G.M.); lucio.caccamo@policlinico.mi.it (L.C.); cristiano.quintini@policlinico.mi.it (C.Q.); 5 Nuclear Medicine Department, Foundation IRCCS Cà Granda Ospedale Maggiore Policlinico, 20122 Milan, Italy; luigia.florimonte@policlinico.mi.it (L.F.); massimo.castellani@policlinico.mi.it (M.C.); 6 School of Speciality General Surgery, University of Milan, 20122 Milan, Italy

**Keywords:** hepatocellular carcinoma, AFP, downstaging

## Abstract

**Background and aims:** Liver transplantation is effective against hepatocellular carcinoma (HCC), but recurrence remains a challenge. Traditional criteria based on tumor size, nodule number, and AFP levels have had limited success in predicting aggressiveness. [18F]FDG PET/CT has shown promise in identifying high-risk tumor features, including microvascular invasion (MVI), which is a key predictor of recurrence. **Methods:** In this retrospective, single-center study, all consecutive patients who underwent LT for HCC between 2010 and 2019 were included. Prior to listing, the patients underwent [18F]FDG PET/CT, and explant pathology was analyzed for MVI and other histological features. The primary objective was to identify the predictors of early HCC recurrence (within 24 months after LT). Secondary objectives included identifying predictors of high-risk histological features of the explant, describing recurrence patterns, and assessing post-recurrence survival. **Results:** The study included 143 patients (median age 59 years [IQR 54–64], 85% males, median MELD 10 [IQR 8–14], median AFP value 8.5 [IQR 4–39] ng/mL) and 40 (28%) with intra-hepatic [18F]FDG PET/CT positivity. HCC recurred post-LT in 25 patients (17%) (median post-LT follow-up 49 months [IQR 28.5–77]) and within 24 months in 12 patients (48%). MVI at the explant stage was independently associated with early recurrence (HR: 7.20, 95% CI 1.82–28.45, *p* = 0.005), while intra-hepatic [18F]FDG PET/CT positivity before LT independently predicted MVI in explants (OR 3.90, 95% CI 1.30–11.71, *p* = 0.01). **Conclusions:** [18F]FDG PET/CT may offer a valuable tool for pre-transplant risk assessment by identifying MVI, which is an independent predictor of early cancer recurrence. Its incorporation into the selection criteria for LT may enhance patient stratification and post-transplant outcomes.

## Simple Summary

Liver transplantation can cure hepatocellular carcinoma (HCC), but some patients still experience cancer recurrence after surgery, especially within the first two years. Accurately identifying patients at higher risk of recurrence before transplantation remains challenging, as standard selection criteria mainly rely on tumor size and number and do not fully reflect tumor aggressiveness. In this study, we evaluated whether a metabolic imaging technique, [18F]FDG PET/CT, could help improve risk assessment before liver transplantation. We analyzed a Western cohort of patients who underwent liver transplantation for HCC and compared PET/CT findings with detailed pathological features of the explanted liver and post-transplant outcomes. We found that tumors showing increased glucose uptake on PET/CT were more likely to display microvascular invasion, a pathological feature strongly associated with early cancer recurrence after transplantation. Importantly, PET/CT positivity indirectly identified patients at higher risk of early recurrence by identifying aggressive tumor biology before surgery. These findings suggest that [18F]FDG PET/CT may provide valuable additional information beyond conventional imaging and could help refine patient selection and post-transplant surveillance strategies in liver transplantation for HCC.

## 1. Introduction

Hepatocellular carcinoma (HCC) remains a leading cause of cancer-related mortality worldwide, with liver transplantation (LT) being a potentially curative option for selected patients [1]. The Milan criteria (MC) have traditionally guided patient selection to ensure favorable post-transplant outcomes [2]. However, using morphological criteria alone may exclude certain patients who could benefit from transplantation. As a result, in recent years, several alternatives and extended criteria have been proposed [3,4,5,6]. Additionally, recent studies have shown that concordance rates between pre-LT imaging and histopathological results are low, especially in patients with multiple tumor nodules and in patients who have undergone previous locoregional treatments [7].

A significant challenge in the refinement of LT criteria is the ability to predict tumor behavior and aggressiveness and thus the risk of recurrence. Although tumor size and number of nodules, assessed through imaging, have been widely used as surrogate markers of tumor burden, they are insufficient for predicting key pathological features such as microvascular invasion (MVI) and tumor differentiation, which strongly influence the risk of recurrence [8]. In this setting, downstaging treatment has emerged as a valuable strategy not only for expanding transplant eligibility but also for providing insight into the biological behavior of tumors. The XXL trial demonstrated that patients with HCC criteria exceeding Milan thresholds who were successfully down-staged benefitted hugely compared to those receiving the standard of care without LT, maintaining a post-transplant survival comparable to those initially within standard criteria, supporting the role of downstaging as a selection tool for liver transplantation [9]. Similarly, retrospective studies have reinforced that downstaging response correlates with favorable outcomes and low recurrence risk [3,10,11]. Also, in relation to the growing need to predict the response to downstaging therapies, various biomarkers and surrogates, including alpha-fetoprotein (AFP), protein induced by vitamin K absence-II (PIVKA-II), and gene expression profiles, have been investigated to improve prognostic accuracy [12,13,14,15,16]. Among these, 18F-fluorodeoxyglucose positron emission tomography/computed tomography ([18F]FDG PET/CT) has emerged as a promising tool, providing metabolic insights into tumor biology and demonstrating a strong association with histopathological findings in explanted livers [17,18,19]. Additionally, [18F]FDG PET/CT has been shown to detect features such as major vessel invasion, serosal involvement, and intra-hepatic metastasis, further supporting its role in risk stratification before LT [20,21].

Finally, early recurrence after LT (occurring within 24 months) remains a significant concern and is associated with worse survival outcomes [21]. In this context, the recent policies of aggressive downstaging, associated with evident limits of association between radiological disease and explant histological disease, can lead to a higher risk of recurrence without adequate predictive and prognostic tools [7].

The aim of our study was to evaluate independent predictors of aggressive tumor histological features and early HCC recurrence after LT, with a particular focus on the role of [18F]FDG PET/CT in improving risk stratification.

## 2. Methods

In this single-center retrospective study, we included all consecutive patients who underwent liver transplantation for hepatocellular carcinoma as the primary indication between 2010 and 2019.

In addition to the examinations required to determine liver transplant eligibility, including multiphasic contrast-enhanced thoracic–abdomen–pelvic CT scans, all patients underwent [18F]FDG PET/CT before listing as per internal protocol to exclude the presence of extrahepatic spread. All acquisitions were performed using a dedicated PET/CT scanner (Biograph True Point 64; Siemens CTI, Knoxville, TN, USA). All patients underwent whole-body PET/CT imaging 50–60 min after intravenous injection of 3.5 MBq/kg of [18F]FDG. Before undergoing the scan, patients were required to fast for a minimum of six hours. Blood glucose levels were verified using a dedicated electronic device to ensure a level < 1.5 g/L. The initial procedure involved the execution of low-dose helical CT (120 kV, 80–100 mAs) for anatomical correlation and attenuation assessment. Subsequently, whole-body emission images were obtained. using 6–7 overlapping bed positions of 2 min each and reconstructed using a line of response-row action maximum likelihood algorithm (2 iterations, 28 subsets, postfilter 5.1 mm) with and without CT attenuation correction (matrix size of 128 × 128, voxel size, 4 × 4 × 4 mm^3^). Intrahepatic [18F]FDG PET/CT positivity was defined as the presence of a focal area of increased radiopharmaceutical uptake within the hepatic lesion that exceeded the surrounding physiological liver activity. A visual analysis of [18F]FDG PET/CT images was performed by two independent observers. In cases of disagreement, the definitive decision was established by collegial consensus. Demographic, clinical, and laboratory data at first HCC diagnosis, at listing, on the day of LT, and upon HCC recurrence were retrospectively collected and analyzed.

The primary objective of the study was to identify predictors at the time of LT for early HCC recurrence, defined as HCC recurrence within the first 24 months after LT. Secondary objectives were to identify clinical, biochemical, or radiological predictors of histological high-risk features of recurrence at explant pathology and to describe the pattern of HCC recurrence after LT and its impact on survival.

The study was approved by the Ethical Committee “Lombardia 3” (study ID 4428_S_P) and complied with the ethical standards and the Helsinki Declaration of 1975, as revised in 2008, and local laws, as well as the European Parliament and Council Regulation (EU) 2016/679 on the protection of natural persons with regard to the processing of personal data, which was enacted on 27 April 2016. Informed consent was waived owing to the retrospective nature of the study, according to Italian law.

### 2.1. Cirrhosis and HCC Diagnosis, Staging and Treatment

Cirrhosis was diagnosed by liver biopsy (stage 4 fibrosis by METAVIR score or stage 5–6 fibrosis according to the Ishak score) or by non-invasive testing (transient elastography [TE] showing liver stiffness greater than 12 kPa) and graded according to the Child–Pugh and/or Model End Stage Liver Disease (MELD) classification [22,23,24]. HCC was diagnosed according to concurrent guidelines and classified according to the Up-to-seven criteria [4]. Bridging treatments to LT, when applied, were defined during multidisciplinary team discussions.

After liver transplantation, all patients underwent surveillance with chest and abdominal contrast-enhanced computed tomography (CT) every 6 months for the first 5 years, after which the surveillance interval was extended to once per year. If HCC recurred within 24 months of LT, early recurrence was considered. Treatment of HCC recurrence depended on the site of recurrence and the multidisciplinary evaluation of each case.

### 2.2. Statistical Analysis

Continuous variables were reported as median and interquartile range (IQR) and compared using Kruskal–Wallis one-way analysis of variance. The Mann–Whitney test was also used when appropriate. Categorical variables were expressed as the number and percentage of cases and compared using the chi-square or Fisher’s exact test when appropriate. The prevalence and incidence of HCC recurrence were reported as percentages. Estimation of HCC incidence was performed using the Kaplan–Meier estimator during a follow-up period considered as the interval between liver transplantation and HCC recurrence, retransplant, death, or last visit. Cox’s proportional hazard model was used to identify variables associated with HCC recurrence. In competing-risk analyses, early recurrence was considered the event of interest, and death prior to early recurrence was treated as a competing event. Baseline variables available before LT and the histological characteristics of explant pathology were evaluated separately using two different predictive models. Logistic regression was used to identify baseline predictors of unfavorable explant pathology. Only variables found to be significant in univariable analysis were included in the multivariable models; *p*-values < 0.05 were considered significant. Results were expressed as hazard ratios (HRs) for Cox analysis and odds ratios (ORs) for logistic regression. The proportional hazards assumption was assessed using Schoenfeld residuals (global and covariate-specific tests). A borderline deviation from proportionality was observed for the variable “Number of nodules at explant”; therefore, a sensitivity analysis including a time-dependent effect (interaction with log(time)) was performed, and the time-varying term was not statistically significant, supporting the robustness of the findings. Overall survival (OS) was measured from the date of LT until the date of death from any cause or the date of the last visit. Data management and analysis were performed using the STATA/SE 12.0 STATA package (Stata Corp., 4905 Lakeway Dr, College Station, TX, USA).

## 3. Results

Between August 2010 and November 2019, 143 patients underwent LT for HCC at our center, and all patients underwent [18F]FDG PET/CT prior to listing. Among them, 124 (87%) underwent [18F]FDG PET/CT within 12 months before liver transplantation and 77 (53%) within 6 months. The patients’ characteristics at the time of LT are reported in Table 1: 122 (85%) were male, the median age was 59 years (54–64), the median MELD score was 10 (8–14), the median AFP value at the time of LT was 8.5 (4–39) ng/mL, and 40 (28%) patients had intra-hepatic [18F]FDG PET/CT positivity. Twenty-three patients tested positive for [18F]FDG PET/CT within 6 months before LT (16%). Among them, the median AFP was 9 (4–47), the median MELD score was 11 (8–15), MVI at explant pathology was present in 11 patients (47.8%), satellite lesions were present in 5 patients (21.7%), median positive nodules on [18F]FDG PET/CT were 1 (1–3), and the median maximum standardized uptake value (SUV) was 4.03 (2.78–5.78). Furthermore, in the positive [18F]FDG PET/CT group, 15 (65%) patients underwent treatment before LT, and in 4 (17%) patients, [18F]FDG PET/CT was performed after the treatment. The characteristics of the patients who underwent [18F]FDG PET/CT within 6 months before LT are shown in Table 2.

Patients were followed up for a median of 49 months (28.5–77) after liver transplantation.

One hundred and nineteen patients (83%) underwent at least one bridge treatment before LT. Among them, 102 (72%) underwent transarterial chemoembolization (TACE), 58 (41.7%) underwent microwave thermoablation (MWTA), 3 (2.1%) underwent transarterial radioembolization (TARE), and 14 (10%) had previously undergone surgical resection.

At the time of analysis (August 2022), 33 (23%) patients had died: 14 (42%) due to HCC recurrence, 5 (15%) due to infections, 3 (9%) due to non-hepatic neoplasia, 1 (3%) due to post-surgery complications, and 9 (27%) due to other non-liver-related causes.

### 3.1. Liver Explant Characteristics

On histological examination, liver cirrhosis was confirmed in most patients (138, 97%). Active HCC nodules were found in 123 (85%) patients, with a median active tumor size of 32.5 mm (20–59) and coexistent microvascular invasion in 39 (27%). The main characteristics of the explanted livers are reported in Table 3.

### 3.2. Post-Transplant HCC Recurrence

**Figure 1:**
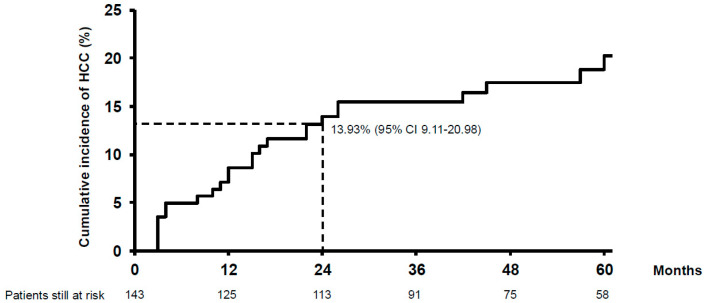
Cumulative post-transplant HCC recurrence. The early recurrence rate (24 months) was 13.93% (95% CI 9.11–20.98).

During a median follow-up time of 49 months (28–77), HCC recurred in 25 (17%) patients, corresponding to a mean annual incidence of 3.99% [95% confidence interval (CI) 2.69–5.90] and a cumulative incidence of 8.62% (95% CI 4.99–14.69) at 12 months and 13.93% (95% CI 9.11–20.98) at 24 months (Figure 1). Nineteen (13.3%) patients experienced early recurrence (within 24 months of liver transplantation). In these patients, the 12-month cumulative incidence of HCC recurrence was 62% (95% CI 42.5–83.4).

Recurrence was extrahepatic in 24 patients (96%), with lung (9.37%) and bone (5.21%) being the most frequent sites of metastatic spread; five patients (21%) had metastases in multiple sites. Only one patient had HCC recurrence that was limited to the liver and developed a single nodule. As for patients with early HCC recurrence, extrahepatic localization was present in 18 patients (95%), with the lung (31%) and bone (21%) as the most frequent sites; three patients (15.7%) had metastases in multiple sites. After the diagnosis of HCC recurrence, 2 (8%) patients underwent surgery and 3 (12%) were treated with sorafenib alone, while 13 (52%) received combination therapy with surgery and systemic treatment, and other combined treatments were offered to 5 patients (20%). Two patients (8%) did not receive active treatment for HCC recurrence.

The overall survival rates were 92% (95% CI 86–96) at 1 year, 86% (95% CI 79–91) at 3 years, and 79% (95% CI 71–85) at 5 years. The overall survival was significantly shorter in patients with HCC recurrence than in HCC-free patients (5-year 38% vs. 89%, *p* < 0.001). Moreover, among patients with HCC recurrence, survival after recurrence was shorter in patients with early recurrence, although the difference was not statistically significant due to the low number of patients in each group [median OS time in months 23 (6–44) vs. not reached (26-not reached) *p* = 0.10].

### 3.3. Predictors of Early HCC Recurrence After LT (<24 Months)

**Figure 2:**
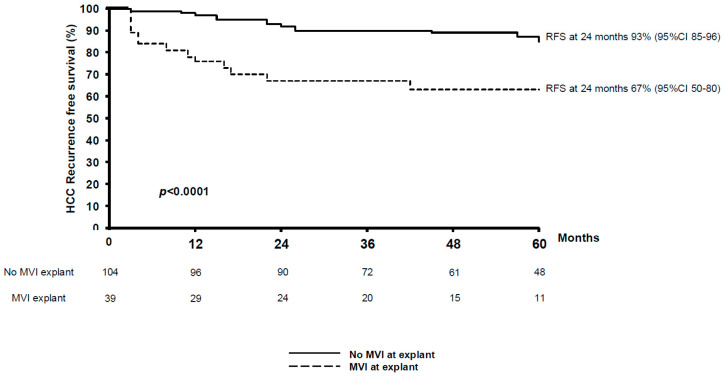
Post-transplant recurrence free survival stratified by presence of microvascular invasion at explant pathology.

In our first predictive model, including only variables available at LT, no independent predictors of post-LT early HCC recurrence were identified (Table 4). In light of the limited number of events and to avoid model overfitting, in the second predictive model, which included explant pathology features, the multivariate analysis was simplified by retaining only the three most clinically relevant covariates [25]: the number of nodules, largest nodule diameter and MVI. The three variables were all predictors of early HCC recurrence in univariate analysis (Table 4), while MVI and largest nodule diameter were the only independent predictors of early HCC recurrence after LT [HR 3.48 (95% CI 1.13–10.71, *p* = 0.030) and HR 1.03 (95% CI 1.00–1.06, *p* = 0.008), respectively]. Patients with MVI at explant pathology had a shorter recurrence-free survival [21 (2–91) months vs. 35 (3–107) months, *p* < 0.001] (Figure 2).

### 3.4. Pre-LT Predictors of MVI at Explant Pathology

In the univariable logistic regression analysis, an intrahepatic positive [18F]FDG PET/CT scan within 6 months before LT and “Up-to-Seven out” HCC at diagnosis were both predictors of MVI at explant pathology, while only a positive [18F]FDG PET/CT scan within 6 months before LT predicted the presence of microvascular invasion by multivariable analysis [OR 3.90 (95% CI 1.30–11.71, *p* = 0.015)] Table 5. In a sensitivity analysis restricted only to patients who underwent [18F]FDG PET/CT prior to locoregional therapy, PET positivity remained significantly associated with microvascular invasion [OR 3.8 (95% CI 1.05–14.21, *p* = 0.042)].

## 4. Discussion

Our study demonstrates that pre-transplant [18F]FDG PET/CT is a significant predictor of microvascular invasion in explanted livers and thus a surrogate marker of aggressive tumor biology, possibly associated with to early HCC recurrence following liver transplantation. These findings suggest that incorporating [18F]FDG PET/CT into pre-transplant assessment could improve patient selection for LT as a surrogate for tumoral biological features. Increased [18F]FDG uptake likely reflects underlying metabolic reprogramming toward enhanced glycolysis and hypoxic adaptation, processes that are commonly associated with poor differentiation, vascular infiltration, and invasive growth patterns. These biological features may explain the observed association between PET positivity, microvascular invasion at explant pathology, and early post-transplant recurrence.

LT represents the optimal therapeutic option for HCC, offering the potential for both the eradication of malignancy and the management of underlying liver disease [2]. However, its clinical application is constrained by the limited availability of donor organs, necessitating stringent selection criteria to prioritize candidates with the highest likelihood of favorable outcomes. Traditional morphologic selection systems, such as the Milan criteria, focus exclusively on tumor size and number, but these parameters have demonstrated clear limitations in predicting post-transplant recurrence [2,4]. Indeed, they fail to account for key histological features such as MVI, tumor differentiation, and satellite nodules, which strongly influence recurrence risk but are only assessable after explant pathology. In our cohort, a positive [18F]FDG PET/CT scan within six months before LT and before bridge treatment was independently associated with the presence of MVI in explant pathology, which is known to be a strong predictor of recurrence, as confirmed in the present study.

In the evolving path to better define LT eligibility criteria, [18F]FDG PET/CT has emerged as a promising tool that can noninvasively capture tumor biology and biochemical aggressiveness preoperatively, thus guiding transplant decision-making in a more personalized and proactive manner [20,26].

This study is the first to explore the utilization of [18F]FDG PET/CT technology in the context of early HCC recurrence after LT in Western countries. The primary objective of our study was to elucidate the association between FDG uptake and histopathological characteristics of tumor aggressiveness, with a particular focus on the early recurrence of HCC. The majority of Asian cohort studies and previous Western studies available in the literature focused on HCC recurrence at any point following LT, which might be influenced by numerous factors beyond the intrinsic characteristics of the tumor [17,20,26,27].

As demonstrated in our study, the presence of MVI in explant pathology was independently associated with an increased risk of early recurrence, defined as recurrence within the first 24 months after LT, reinforcing findings from other studies that highlight the prognostic importance of MVI in other curative HCC treatments such as surgical resection [8].

Despite its potential [28], the systematic use of [18F]FDG PET/CT in HCC staging has faced criticism for its cost. A recent study by Nault et al., using a large French multicenter dataset, found that incorporating [18F]FDG PET/CT into routine pre-treatment staging of HCC was not cost-effective in altering therapeutic decision-making for the general HCC population [29]. The authors concluded that, given the high cost and limited impact on treatment allocation, [18F]FDG PET/CT should not be systematically integrated into HCC work-up outside of specific clinical indications.

However, our findings suggest a potential redefinition of the role of [18F]FDG PET/CT in a more targeted context. Given the critical prognostic weight of early recurrence, which is associated with significantly impaired post-LT survival, incorporating [18F]FDG PET/CT into the pre-transplant staging algorithm could enhance the identification of patients with histological characteristics that increase the risk of recurrence despite morphologically acceptable disease at imaging. Although not universally cost-effective across all treatment settings, [18F]FDG PET/CT may have clinical utility in refining transplant eligibility and surveillance strategies within a specific subgroup of patients evaluated for LT. Future prospective cost–benefit analyses should consider this more nuanced application when evaluating the utility of [18F]FDG PET/CT in HCC.

This could be particularly useful in the downstaging algorithm applied to LT, which allows patients with HCC initially outside the traditional transplant criteria to still benefit from LT after responding to aggressive treatment. As downstaging becomes a more common approach in LT for HCC, integrating [18F]FDG PET/CT into the decision-making process could improve the efficacy of these strategies and reduce the risk of recurrence after LT. The 2024 EASL guidelines introduced structured and dynamic downstaging criteria, moving beyond purely morphological limits to include biological and temporal dimensions of tumor behavior [1]. Notably, after successful downstaging, the guidelines suggest that patients maintain a stable radiologic response and an AFP level ≤ 1000 ng/mL for at least 3 months before being considered for transplantation. This interval acts as a biological “stress test”, reflecting the underlying tumor biology and helping select patients with more favorable outcomes. In this evolving framework, [18F]FDG PET/CT may offer additional value by identifying metabolically aggressive tumors before transplantation, serving as a noninvasive surrogate for histological features such as microvascular invasion and poor differentiation, which are otherwise only assessable post-explant [3,4,30].

Although our study presents promising results, some limitations should be acknowledged. The retrospective single-center design of our study may limit the generalizability of our findings. Despite restricting analyses to [18F]FDG PET/CT performed within 6 months before transplantation and prior to locoregional therapy, some residual heterogeneity in imaging timing may have influenced FDG uptake.

Additionally, although we found a strong association between [18F]FDG PET/CT and MVI, its ability to predict late recurrence beyond the first two years remains unclear. Future prospective multicenter studies and larger cohorts will be essential to validate these findings and further explore the role of [18F]FDG PET/CT in predicting both early and late HCC recurrence.

In conclusion, [18F]FDG PET/CT enhances HCC risk assessment before liver transplantation by providing a metabolic snapshot of HCC biology and predicting key factors, such as MVI, leading to better patient selection and tailored post-transplant monitoring.

## 5. Conclusions

Pre-transplant [18F]FDG PET/CT identifies aggressive tumor biology in patients undergoing liver transplantation for hepatocellular carcinoma by predicting microvascular invasion at explant pathology. Through this association, PET/CT positivity indirectly identifies patients at increased risk of early post-transplant recurrence (<24 months) and may support improved risk stratification and tailored post-transplant management.

## Figures and Tables

**Table 1:** Patients’ pre-LT characteristics.

Characteristic	All Patients (N = 143)	Without Early HCC Recurrence (N = 124)	With Early HCC Recurrence (N = 19)	*p*-Value
Age, years, median (IQR)	59 (54–64)	60 (41–70)	57 (48–74)	0.046
Males, N (%)	122 (85)	20 (95.2)	1 (4.7)	0.308
BMI, kg/m^2^, median (IQR)	25 (23–29)	25 (17–40)	30 (20.6–38)	0.213
Active smoker, N (%)	39/128 (30)	33/113 (29.2)	6/15 (40)	0.727
Etiology				0.95
Viral	104 (73)	89 (85.6)	15 (14.4)	
MASLD	9 (6)	7 (77.8)	2 (22.2)	
ALD	21(15)	19 (90.5)	2 (9.5)	
Other	9 (6)	9 (100)	0 (0)	
Median largest nodule’s diameter (radiologic), mm, median (IQR)	20 (4–45)	19 (4–45)	20 (10–40)	0.115
Nodule number (radiology), median (IQR)	2 (1–8)	2 (1–8)	3.5 (1–4)	0.086
Up-to-seven out at diagnosis, N (%)	10/139 (7)	8 (6.6)	2 (11.1)	0.618
HCC treatment before LT, N (%)	119/140 (85)	103/122 (84.4)	16/18 (88.9)	0.470
Number of treatments, median (IQR)	2 (1–3)	2 (1–3)	2.5 (2–4)	0.209
Best response to treatment before listing				0.320
Complete response, N (%)	21/135 (15)	17/117 (14.5)	4/18 (22.2)	
Partial response, N (%)	69/135 (51)	58/117 (49.5)	11/18 (61.1)	
Stable disease, N (%)	20/135 (15)	18/117 (15.3)	2/18 (11.1)	
Progression, N (%)	25/135 (19)	24/117 (20.5)	1/18 (5.5)	
Up-to-seven out at listing, N (%)	4/136 (3)	4/118 (3.4)	0/24 (0)	1.000
Median time between last HCC treatment and LT, months, median (IQR)	5 (1–9)	5 (2–9)	5 (1–8.5)	0.773
Positive [18F]FDG PET/CT scan before LT, N (%)	40 (28)	33 (26.6)	7 (36.8)	0.412
Median time between FDG PET scan and LT, months, median (IQR)	6 (4–9)	6 (4–10)	5 (3–7)	0.222
Median MELD at LT, median (IQR)	10 (8–14)	10 (8–14)	10 (8–13)	0.902
CTP at LT, N (%)				0.194
A	88/143 (62)	75/124 (60.5)	13/25 (68.4)	
B	38/143 (27)	35/124 (28.2)	3/25 (15.8)	
C	16/143 (11)	14/124 (11.3)	3/25 (15.8)	
GGT at LT, U/L, median (IQR)	67 (37–127)	67.5 (37.5–127.5)	67 (37–93)	0.602
Albumin at LT, mg/dL, median (IQR)	3.9 (3.4–4.2)	3.9 (3.4–4.2)	4 (3.5–4.4)	0.450
Platelet count at LT (×10^9^/L), median (IQR)	74 (47–117)	74 (46–116)	81 (58–117)	0.50
AFP at LT, ng/dL, median (IQR)	8.5 (4–39)	8 (4–39)	12 (7–54)	0.198
AFP > 7 ng/dL, N (%)	76/142 (53)	64/123(52)	12/19 (63.1)	0.461
AFP > 200 ng/dL, N (%)	10/142 (7)	8/123 (6.5)	2/19 (10.5)	0.624
AFP > 1000 ng/dL, N (%)	4/142 (3)	3/123 (2.4)	1/19 (5.2)	0.441

BMI: body mass index; MASLD: metabolic-associated liver disease; ALD: alcohol-related liver disease; HCC: Hepatocellular carcinoma; LT: liver transplant; [18F]FDG PET/CT: positron emission tomography and computed tomography; MELD: model for end-stage liver disease; CTP: Child–Turcotte–Pugh; GGT: gamma-glutamyl transpeptidase; PLT: platelets; AFP: alpha-fetoprotein.

**Table 2:** Patients’ characteristics according to [18F]FDG PET/CT results.

Characteristic	[18F]FDG PET/CT Positive (n = 23)	[18F]FDG PET/CT Negative (n = 54)	*p*-Value
Age, years, median (IQR)	59 (54–64)	58 (53–63)	0.472
Males, N (%)	19 (27.1)	51 (72.9)	0.187
Median largest nodule’s diameter (radiologic), mm, median (IQR)	16 (11–21)	20 (13–25)	0.218
Nodule number (radiology), median (IQR)	1 (1–3)	2 (1–3)	0.120
Up-to-seven out at diagnosis, N (%)	2 (66.7)	1 (33.3)	0.209
Microvascular invasion at explant, N (%)	11 (47.8)	11 (20.4)	0.026
Satellite lesions at explant, N (%)	5 (21.7)	6 (11.1)	0.288
HCC treatment before LT, N (%) Number of treatments, median (IQR)	115/23 (25.4) 2 (0–4)	44/53 (74.6) 2 (1–3)	0.132 0.887
Median MELD at LT, median (IQR)	11 (8–15)	11 (9–14)	0.688
CTP at LT, N (%)			0.938
A	13/22 (59)	27/54 (50)	
B	6/22 (27.2)	19/54 (35.2)	
C	3/22 (13.6)	11/54 (20.4)	

HCC: hepatocellular carcinoma; LT: liver transplant; [18F]FDG PET/CT: positron emission tomography and computed tomography; MELD: model for end-stage liver disease; CTP: Child–Turcotte–Pugh.

**Table 3:** Histological characteristics of explanted livers.

Explant Pathology	All Patients (n = 143)	Without Early HCC Recurrence (n = 124)	With Early HCC Recurrence (n = 19)	*p*-Value
Diagnosis of cirrhosis, N (%)	137/141 (97)	119/123 (96.7)	18/18 (100%)	1.000
Number of nodules, median (IQR)	2 (1–4)	2 (1–4)	3 (2–5)	0.043
Total nodule size, mm, median (IQR)	42 (23–74)	38.5 (22–63)	86 (59–100)	<0.001
Largest nodule diameter, mm, median (IQR)	24 (17–32)	23.5 (17–30)	33.5 (25–60)	0.010
Active nodules, N (%)				0.139
0	20 (14)	18 (14.5)	2 (10.5)	
1	46 (32)	43 (34.7)	3 (15.8)	
2–3	47 (33)	40 (32.2)	7 (36.9)	
>3	30 (21)	23 (18.4)	7 (36.8)	
Total active nodule size, mm, median (IQR)	25 (11–54)	28 (18–53)	56 (52–88)	0.002
Largest active nodule diameter, mm, median (IQR)	18 (10–28)	18 (10–25)	28 (12–40)	0.037
Presence of satellites, N (%)	23 (16)	16 (12.9)	7 (36.8)	0.016
Microvascular invasion, N (%)	39 (27)	27 (21.7)	12 (63.1)	<0.001
Milan-in, N (%)	87 (61)	82 (66.1)	5 (26.3)	0.002

HCC: hepatocellular carcinoma.

**Table 4:** Predictors of early HCC recurrence after LT (within the first 24 months).

		Univariable Analysis			Multivariable Analysis		
Variable	Type of Variable	HR	95% CI	*p*-Value	HR	95% CI	*p*-Value
**Clinical and Radiological Variables**							
Males	Yes vs. no	0.31	0.41–2.31	0.254			
Age, years	Continuous	0.94	0.88–1.01	0.092			
BMI, kg/m^2^	Continuous	1.07	0.97–1.18	0.125			
Active smoker at LT	Yes vs. no	1.23	0.69–2.19	0.474			
Etiology (Viral)	Yes vs. no	0.70	0.43–1.16	0.167			
MELD score	Continuous	1.02	0.94–1.12	0.595			
CPT	A/B vs. C	0.92	0.71–1.19	0.510			
Albumin, g/dL	Continuous	1.42	0.63–3.22	0.392			
PLT, n/mm^3^	Continuous	1.00	0.99–1.00	0.815			
AFP > 1000 ng/mL	Yes vs. no	1.99	0.26–14.96	0.501			
Up-to-Seven out at diagnosis	Yes vs. no	1.62	0.37–7.06	0.518			
Complete response to HCC bridge treatment	Yes vs. no	2.39	0.94–6.09	0.066			
Positive [18F]FDG PET/CT within 6 months before LT	Yes vs. no	1.38	0.46–4.14	0.557			
Largest nodule’s diameter (radiologic), cm	Continuous	1.03	0.94–1.12	0.470			
Number of nodules (radiologic)	Continuous	1.29	0.87–1.91	0.206			
**Liver Explant Variables**							
Number of nodules	Continuous	1.08	1.00–1.16	0.035	1.06	0.97–1.15	0.146
Largest nodule’s diameter, cm	Continuous	1.04	1.01–1.06	0.001	1.03	1.00–1.06	0.008
Microvascular invasion	Yes vs. no	5.34	2.10–13.59	<0.001	4.26	1.56–11.61	0.005

BMI: body mass index; MELD: model for end-stage liver disease; CTP: Child–Turcotte–Pugh; PLT: platelets; AFP: alpha-fetoprotein; HCC: hepatocellular carcinoma; [18F]FDG PET/CT: positron emission tomography and computed tomography; LT: liver transplant.

**Table 5:** Pre-transplant predictors of microvascular invasion at explant histology.

		Univariable Analysis			Multivariable Analysis		
Variable	Type of Variable	OR	95% CI	*p*-Value	OR	95% CI	*p*-Value
Males	Yes vs. no	1.07	0.38–3.01	0.885			
Age, years	Continuous	0.99	0.94–1.05	0.931			
BMI, kg/m^2^	Continuous	1.05	0.97–1.14	0.170			
Active smoker at LT	Yes vs. no	0.77	0.48–1.24	0.285			
Etiology (Viral)	Yes vs. no	0.67	0.44–1.02	0.066			
MELD score	Continuous	1.03	0.96–1.11	0.309			
CPT B or C	Yes vs. No	1.05	0.87–1.27	0.561			
Albumin, g/dL	Continuous	0.70	0.36–1.37	0.307			
PLT, n/mm^3^	Continuous	0.99	0.99–1.00	0.738			
AFP > 1000 ng/mL	Yes vs. no	8.5	0.85–84.34	0.068			
Up-to-Seven in at diagnosis	Yes vs. no	4.54	1.20–17.13	0.025	3.72	0.27–49.74	0.321
Milan’s criteria in at enlisting	Yes vs. no	0.50	1.89–1.35	0.174			
Complete response to HCC bridge treatment	Yes vs. no	0.46	0.16–1.31	0.149			
Positive [18F]FDG PET/CT within 6 months before LT	Yes vs. no	3.58	1.25–10.26	0.017	3.90	1.30–11.71	0.015
Largest nodule’s diameter (radiologic), cm	Continuous	0.98	0.92–1.04	0.613			
Nodule number (radiologic)	Continuous	1.09	0.78–1.53	0.598			

BMI: body mass index; MELD: model for end-stage liver disease; CTP: Child–Turcotte–Pugh; PLT: platelets; AFP: alpha-fetoprotein; HCC: hepatocellular carcinoma; [18F]FDG PET/CT: positron emission tomography and computed tomography; LT: liver transplant.

## Data Availability

Data are available upon reasonable request to the corresponding author.

## Abbreviations

Hepatocellular carcinoma (HCC); liver transplant (LT); Milan criteria (MC); microvascular invasion (MVI); alpha-fetoprotein (AFP); protein induced by vitamin K absence-II (PIVK-II); 18F-fluorodeoxyglucose positron emission tomography/computed tomography ([18F]FDG PET/CT); Transient Elastography (TE); model end-stage liver disease (MELD); computed tomography (CT); interquartile range (IQR); hazard ratios (HRs); overall survival (OS); odds ratios (ORs); trans-arterial chemoembolization (TACE); microwave thermoablation (MWTA); transarterial radioembolization (TARE); confidence interval (CI).
